# Clinical and Symptom Profiles of Long-COVID Patients in Italy: A Cross-Sectional Analysis

**DOI:** 10.3390/healthcare13212706

**Published:** 2025-10-27

**Authors:** Angelo Cianciulli, Emanuela Santoro, Roberta Manente, Antonietta Pacifico, Ilaria Barberio, Vittoria Satriani, Giovanni Boccia

**Affiliations:** 1 Department of Medicine, Surgery and Dentistry ‘’Scuola Medica Salernitana”, University of Salerno, 84081 Salerno, Italy; ancianciulli@unisa.it (A.C.); apacifico@unisa.it (A.P.); ilabarb95@gmail.com (I.B.); vsatriani@unisa.it (V.S.); gboccia@unisa.it (G.B.); 2 San Giovanni di Dio e Ruggi d’Aragona University Hospital, 84081 Salerno, Italy; manente392@gmail.com; 3 Integrated Care Department of Health Hygiene and Evaluative Medicine, San Giovanni di Dio e Ruggi d’Aragona University Hospital, 84131 Salerno, Italy; 4 Hospital and Epidemiological Hygiene Unit, San Giovanni di Dio and Ruggi D’Aragona University Hospital, 18 Hospital, 84131 Salerno, Italy

**Keywords:** Long COVID, persistent symptoms, Italy, nursing, healthcare policy, short communication

## Abstract

**Background/Objectives**: Long COVID is a multisystemic condition persisting beyond the acute phase of SARS-CoV-2 infection. Data on young, community-dwelling adults in Italy remain limited. To describe the sociodemographic, clinical, and symptom profiles of Long-COVID patients in an Italian cohort. **Methods**: Cross-sectional survey (February–April 2025) on 250 adults with prior COVID-19. A validated 24-item questionnaire was administered. Descriptive statistics, 95% confidence intervals (CIs)**,** Hedges’ g effect sizes, and exploratory subgroup analyses (sex, age ≤ 30 vs. >30) were performed. **Results**: Participants were 63.6% female, 56% ≤ 30 years, 4.4% with comorbidities. Acute symptoms included muscle/joint pain (2.79 ± 1.31), weakness (2.77 ± 1.28), and tiredness (2.76 ± 1.31). Persistent symptoms were excessive tiredness (2.36 ± 1.27), weakness (2.25 ± 1.29), and muscle/joint pain (2.25 ± 1.25). Acute → persistent changes were significant (*p* < 0.01, paired *t*-test) with effect sizes g = 0.31–0.42. Women reported higher persistent fatigue (mean diff = 0.40, 95% CI 0.01–0.78, *p* = 0.04). **Conclusions**: Even among young adults without comorbidities, Long COVID imposes a relevant burden. Findings highlight the need for multidisciplinary pathways, nursing-led follow-up, and targeted self-management education.

## 1. Introduction

The World Health Organization (WHO) defines post-COVID-19 condition (Long COVID) as the persistence or new onset of symptoms beyond 12 weeks after acute infection, not explained by alternative diagnoses [1]. Common manifestations include fatigue, dyspnea, musculoskeletal pain, cognitive impairment, and mental health disturbances [2,3,4]. Meta-analyses suggest that up to 80% of patients experience at least one persistent symptom [2,5,6,7,8,9].

In Italy, recent studies have highlighted the impact of Long COVID on quality of life and work capacity [10], but most evidence derives from hospital-based or multicenter cohorts, while data on community-dwelling and younger adults remain limited [9,11].

This study aims to describe the clinical and symptomatological profile of an Italian cohort of Long-COVID patients using a validated questionnaire, to provide insights useful for guiding clinical follow-up and nursing care strategies.

## 2. Materials and Methods

### 2.1. Study Design

A cross-sectional survey was conducted in southern Italy between February and April 2025.

### 2.2. Participants

The study involved 250 adults aged 18 years and older, all with a previous confirmed SARS-CoV-2 infection and persistent symptoms attributable to post-COVID. Acute disease severity was assessed through hospitalization during the acute infection and need for supplemental oxygen. Only 3.6% of respondents reported hospitalization, and 2.8% required oxygen therapy, indicating that the cohort largely represented community-managed, mild-to-moderate cases. Subjects with severe chronic comorbidities or who were unable to complete the questionnaire due to cognitive or linguistic difficulties were excluded. Comorbidities were self-reported and included hypertension, asthma, diabetes, and thyroid disorders. Overall, 4.4% (95% CI 1.9–6.9) of participants reported at least one chronic condition, most commonly hypertension. Only individuals whose symptoms persisted for at least 12 weeks after the acute SARS-CoV-2 infection were included, in accordance with the WHO definition of post-COVID-19 condition. Participants who had recovered within 12 weeks were excluded. The mean time elapsed since the acute infection was 21.3 ± 5.8 weeks (range 12–38 weeks). Information on vaccination status and reinfection history was collected by self-report. Most participants (78%) had received at least one dose of a COVID-19 vaccine before infection, and 12% reported a documented reinfection. Data were collected between February and April 2025, a period in which Omicron subvariants (XBB and JN.1 lineages) were the predominant circulating strains in Italy. The sample size was calculated using the single-proportion formula: *n* = Z^2^ × *p* × (1 − *p*)/*d*^2^, where Z is the standard normal deviate corresponding to the desired confidence level (1.96 for 95%), *p* is the expected prevalence of persistent post-COVID symptoms, and *d* is the desired margin of error. Assuming *p* = 0.50 (maximum variability) and *d* = 0.05, the minimum required sample was *n* = 196. To account for an expected 20% non-response rate and to ensure adequate precision (95% CI within ±6% for the prevalence of any persistent symptom), a final target of 250 participants was set based on feasibility during the study period. Participants were recruited through online announcements and direct invitations to community-based individuals with previously confirmed COVID-19 infection. Data were collected anonymously between February and April 2025. The response rate was approximately 82% (250/305). No systematic differences were observed between responders and non-responders in age or sex distribution.

### 2.3. Instrument

A validated questionnaire consisting of 24 questions was used, divided into three sections: the first dedicated to socio-demographic data, the second to the clinical course of COVID-19 infection, and the third to psychological and physical conditions in the period following the illness. Symptom intensity was assessed using a Likert scale from 0 to 4.

The questionnaire was developed and psychometrically validated by the same research group in a previous study [10], which confirmed its reliability and construct validity in the Italian population. Part of the present dataset was used for that validation analysis. The current Short Communication represents the first real-world application of the validated Long-COVID questionnaire, focusing on the clinical and symptomatological profiles of participants, which were not previously analyzed in the validation paper.

For the assessment of acute-phase symptoms, participants were asked to retrospectively recall their symptom intensity during the initial SARS-CoV-2 infection, using the same Likert scale and identical list of items adopted for the persistent phase. This approach ensured direct comparability between acute and post-acute data. Although recall bias cannot be fully excluded, the relatively short time elapsed since infection (mean 21.3 ± 5.8 weeks) and the use of standardized, closed-ended questions helped minimize its potential effect.

Only the subset of variables used for the psychometric validation (e.g., internal consistency, construct validity, factor structure) overlapped with the previous publication [10]. All analyses presented here are original and focus exclusively on clinical, epidemiological, and symptom-based outcomes.

The questionnaire included distinct items for “muscle weakness”, “fatigue/asthenia”, and “excessive tiredness”, each referring to separate constructs as specified in the validated instrument. Exact item wording is provided in Supplementary Table S1 to ensure conceptual transparency.

### 2.4. Statistical Analysis

Descriptive data analysis was performed by reporting frequencies and percentages, means with standard deviation, and 95% confidence intervals. To assess the variation between the acute and persistent phases, paired *t*-tests were used after verifying normality with the Shapiro–Wilk test; the Wilcoxon test was also applied as a sensitivity analysis. The effect size was estimated using Hedges’ g index corrected for paired data. Subgroup analyses were also conducted based on gender and age (≤30 years vs. >30 years), using *t*-tests and χ^2^ tests. The age threshold of 30 years was selected a priori to distinguish younger adults—representing the majority of community-based post-COVID cases in Italy—from older participants with potentially different comorbidity and exposure profiles. Sensitivity analyses using age as a continuous variable did not materially alter the direction or significance of the results. Statistical significance was set at *p* < 0.05, and 95% confidence intervals were reported for the main results. Missing data were very limited (<5%), and analyses were performed only on complete cases, without resorting to imputation. A per-item analysis of missingness showed that item-level missing values ranged from 0% to 2%, with no systematic pattern across variables. The paired N used for all acute–persistent comparisons was 250, confirming complete data availability for the core symptom set analyzed (Supplementary Table S2). Subgroup comparisons were treated as exploratory. Given the descriptive and hypothesis-generating design of this study, no formal correction for multiple comparisons (e.g., Holm–Bonferroni) was applied, as this could excessively penalize the identification of clinically relevant trends. To support interpretability and mitigate type I error inflation, effect sizes (Hedges’ g) were reported alongside *p*-values. Effect size magnitudes were interpreted according to conventional thresholds for paired-sample designs: small (g = 0.20–0.49), moderate (g = 0.50–0.79), and large (g ≥ 0.80). Paired mean changes (Δ) with SD and 95% CIs were also reported for each symptom.

### 2.5. Ethical Considerations

The study was conducted in compliance with the ethical standards set by the Declaration of Helsinki and with EU Regulation 2016/679 (GDPR). Ethical approval was granted by the Territorial Ethics Committee Campania 2 (protocol no. 2025/3339, 5 February 2025), which ensured that all procedures were carried out in a manner prioritizing participants’ safety, privacy, and autonomy. Written informed consent was obtained from all participants prior to inclusion in the study, after receiving clear information about objectives, confidentiality, and voluntary participation.

## 3. Results

### 3.1. Sociodemographic Characteristics

Among 250 participants, 159 were women (63.6%, 95% CI 57.6–69.6) and 140 were aged ≤ 30 years (56.0%, 95% CI 49.8–62.2) (Table 1).

Comorbidities were assessed by self-report and included hypertension, asthma, diabetes, and thyroid disorders. Overall, 4.4% (95% CI 1.9–6.9) of participants reported at least one chronic condition, most commonly hypertension (Table 2).

### 3.2. Acute-Phase Symptoms

The most frequent acute symptoms were musculoskeletal pain, weakness, and excessive tiredness (Table 3).

### 3.3. Persistent Post-COVID Symptoms

**Figure 1:**
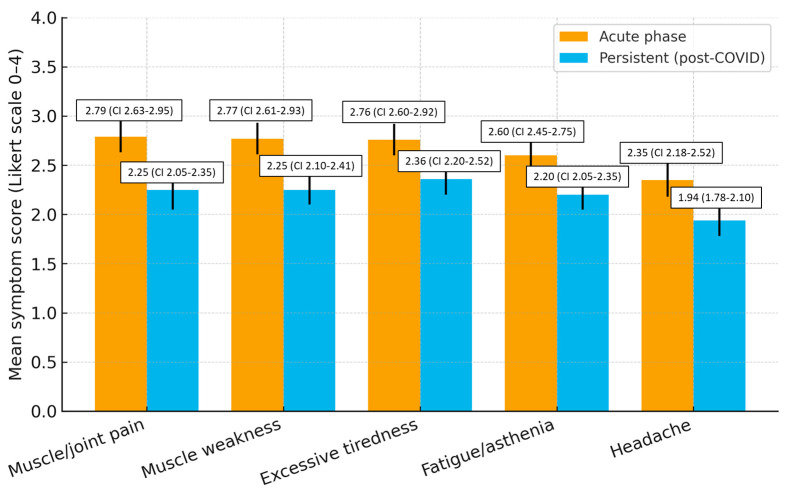
Comparison of mean symptom intensity scores (Likert 0–4) between the acute and persistent (post-COVID) phases (N = 250). Error bars represent 95% confidence intervals derived from Table 2 and Table 3.

Symptoms declined but remained clinically relevant. Compared with the acute phase, mean scores decreased by 0.43 points for excessive tiredness, 0.52 for muscle weakness, 0.54 for muscle/joint pain, 0.40 for fatigue/asthenia, and 0.41 for headache, indicating a small-to-moderate reduction but persistent clinical relevance (Figure 1).

An exploratory inter-item correlation analysis among excessive tiredness, fatigue/asthenia, and muscle weakness revealed moderate correlations (r = 0.48–0.56), indicating that these fatigue-related items represent related but distinct constructs, consistent with the instrument’s validated structure (Table 4).

### 3.4. Symptom Change and Effect Sizes

All symptoms showed small-to-moderate reductions from acute to persistent phase (Table 5).

### 3.5. Subgroup Analyses

Sex: Women reported higher persistent fatigue (mean 2.35 ± 1.20 vs. 1.95 ± 1.28; absolute difference = 0.40, 95% CI 0.01–0.78; *p* = 0.04). Although statistically significant, the difference was small in magnitude and may attenuate after adjustment for potential confounders.

Age: Participants aged ≤ 30 and >30 years showed comparable symptom persistence (all *p* > 0.05).

## 4. Discussion

This Italian cohort demonstrates that young, low-comorbidity adults are not spared from persistent Long-COVID symptoms. Fatigue, weakness, and musculoskeletal pain were most common, consistent with international findings [2,3,7,8,9,12,13,14]. Although the mean symptom reductions (Δ = 0.40–0.54 on a 0–4 Likert scale) corresponded to small-to-moderate effect sizes, no established minimal important difference (MID) currently exists for this questionnaire. Therefore, the clinical significance of these changes remains uncertain and should be interpreted as reflecting modest but measurable symptom improvement rather than full recovery. Italian data [10] and global meta-analyses [8,12] confirm fatigue prevalence ~50–65% up to 6 months. Our results extend evidence to community-based, young adults, a population often underrepresented. Novelty: Unlike hospital cohorts [11,12], our sample captures milder acute infections but still shows substantial sequelae. Female sex emerged as a risk factor for persistent fatigue, in line with UK registry findings [9]. However, this association should be interpreted with caution, as potential confounders such as differences in health-seeking behavior, employment status, caregiving burden, and mental health could partly explain the observed sex effect. Moreover, the marginal *p*-value (*p* = 0.04) might attenuate with adjustment or multiplicity correction. The age-based subgrouping (≤30 vs. >30 years) was selected to reflect population-level differences in exposure and comorbidity, and sensitivity checks treating age as a continuous variable confirmed the robustness of observed patterns.

Limitations include the cross-sectional design, reliance on self-reported data, and absence of biomarker or functional measures. However, the use of a validated instrument [6], reporting of effect sizes, and internal consistency between acute and persistent symptom scoring enhance robustness and interpretability. Acute symptom data were collected retrospectively using identical items and scales, ensuring comparability but potentially introducing recall bias, which was mitigated through standardized question framing and a relatively short recall interval. Subgroup comparisons were exploratory and should be interpreted cautiously.

Recent literature has increasingly interpreted Long COVID through a syndemic perspective, emphasizing the synergistic interaction between biological, psychological, and social determinants of health that exacerbate disease burden. From this standpoint, Long COVID is not merely a post-viral condition but the outcome of complex interplays among comorbidities, psychosocial stressors, and socioeconomic inequities influencing both the course of illness and recovery trajectories. This conceptual framework reinforces the need for integrated, multidisciplinary strategies that combine medical management, psychosocial support, and community-based care. In this context, the role of nurses and community health professionals is pivotal in addressing the social and behavioral factors shaping the long-term impact of COVID-19. This view is consistent with recent evidence which underlines the importance of syndemic thinking in framing COVID-19 as both a biological and a social phenomenon [15,16].

Generalizability: Results apply primarily to younger, community-dwelling Italians with low comorbidity. Findings should therefore be interpreted cautiously and may not extend to older, hospitalized, or multimorbid populations, given the non-probabilistic sampling and community-based setting. The non-probabilistic sampling and low comorbidity rate suggest a possible selection bias toward younger and healthier respondents. Additionally, most participants were vaccinated and infected during the Omicron-dominant period, which may limit comparability with cohorts affected by earlier variants.

### 4.1. Implications for Healthcare Practice and Policy

Clinical practice: Routine monitoring of fatigue, weakness, and pain should be integrated into follow-up visits. Nursing-led interventions focusing on symptom management, energy conservation, and rehabilitation are essential.

Public health: Findings highlight the importance of embedding Long-COVID care within primary care and community nursing services, rather than limiting it to hospital-based clinics. In this framework, environmental and occupational safety—including control of air quality and microclimatic conditions in healthcare settings—represents a complementary dimension of post-pandemic resilience and infection prevention [17].

Policy: Results are consistent with NICE guidelines on Long COVID [18] and WHO recommendations on post-COVID-19 condition [1]. They also resonate with Italian health reforms (DM 77/2022), which emphasize the role of family and community nurses in territorial healthcare models [19].

Research: Future longitudinal multicenter studies are needed to better characterize predictors, clinical trajectories, and long-term outcomes of Long COVID in the Italian population.

### 4.2. Strengths and Limitations

This study has several strengths. First, it used a validated questionnaire specifically designed for the assessment of post-COVID-19 symptoms, enhancing the reliability of data collection. Second, the focus on a community-based and relatively young cohort fills an important gap in the literature, as most studies have concentrated on hospitalized or older populations. Third, the reporting of effect sizes and confidence intervals improves the interpretability and robustness of the findings.

However, limitations should be acknowledged. The cross-sectional design prevents causal inference and limits the ability to track symptom trajectories over time. The non-probabilistic sampling strategy reduces generalizability, while reliance on self-reported measures may introduce recall bias, particularly for acute-phase symptoms. Nevertheless, the use of standardized items, identical measurement scales, and the relatively short recall period help mitigate this potential limitation. Finally, the absence of biomarker data or objective functional assessments restricts clinical characterization.

## 5. Conclusions

Long COVID exerts a significant multidimensional burden even in young adults without prior comorbidities. Persistent symptoms such as fatigue, pain, and headache impair quality of life and call for integrated multidisciplinary approaches. From a syndemic perspective, Long COVID should be viewed not only as a post-viral condition but as the result of interactions between biological, psychological, and social determinants that jointly shape vulnerability, recovery, and long-term outcomes. Recognizing these interconnections underscores the importance of community-based, equity-oriented strategies and reinforces the central role of nurses in addressing the social and behavioral dimensions of care. Longitudinal multicenter studies are warranted to better understand the trajectory of post-COVID symptoms and to optimize supportive care.

## Figures and Tables

**Table 1:** Sociodemographic characteristics of the cohort (N = 250).

Variable	n (%)	95% CI	Missing n (%)
Age ≤ 30 years	140 (56.0)	49.8–62.2	0 (0)
Age > 30 years	110 (44.0)	37.8–50.2	0 (0)
Female	159 (63.6)	57.6–69.6	0 (0)
Male	91 (36.4)	30.4–42.4	0 (0)

**Table 2:** Clinical characteristics of participants (N = 250).

Variable	n (%)	95% CI	Missing n (%)
Comorbidities	11 (4.4)	1.9–6.9	0 (0)
Hospitalization during acute COVID-19	9 (3.6)	1.3–5.9	0 (0)
Oxygen therapy required	7 (2.8)	0.9–4.7	0 (0)

**Table 3:** Symptoms during the acute phase of SARS-CoV-2 infection.

Symptom	Mean ± SD	95% CI	Missing n (%)
Muscle/joint pain	2.79 ± 1.31	2.63–2.95	0 (0)
Muscle weakness	2.77 ± 1.28	2.61–2.93	0 (0)
Excessive tiredness	2.76 ± 1.31	2.60–2.92	0 (0)
Fatigue/asthenia	2.60 ± 1.17	2.45–2.75	0 (0)
Headache	2.35 ± 1.36	2.18–2.52	0 (0)

**Table 4:** Persistent post-COVID symptoms.

Symptom	Mean ± SD	95% CI	Missing n (%)
Excessive tiredness	2.36 ± 1.27	2.20–2.52	0 (0)
Fatigue/asthenia	2.20 ± 1.24	2.05–2.35	0 (0)
Muscle weakness	2.25 ± 1.29	2.10–2.41	0 (0)
Muscle/joint pain	2.25 ± 1.25	2.05–2.35	0 (0)
Headache	1.94 ± 1.26	1.78–2.10	0 (0)

**Table 5:** Acute Persistent change in symptoms (paired analysis, N = 250).

Symptom	Mean (Acute) ± SD	Mean (Persistent) ± SD	Δ (Mean Change) ± SD	95% CI of Δ	Hedges’ g (95% CI)	*p*-Value	Interpretation
Muscle/joint pain	2.79 ± 1.31	2.25 ± 1.25	0.54 ± 1.02	0.37–0.71	0.42 (0.24–0.60)	<0.01	Moderate
Muscle weakness	2.77 ± 1.28	2.25 ± 1.29	0.52 ± 0.98	0.36–0.69	0.40 (0.23–0.58)	<0.01	Moderate
Excessive tiredness	2.76 ± 1.31	2.36 ± 1.27	0.40 ± 0.94	0.25–0.56	0.31 (0.13–0.49)	<0.01	Small
Fatigue/asthenia	2.60 ± 1.17	2.20 ± 1.24	0.40 ± 0.91	0.25–0.55	0.33 (0.16–0.51)	<0.01	Small
Headache	2.35 ± 1.36	1.94 ± 1.26	0.41 ± 0.88	0.26–0.56	0.31 (0.14–0.49)	<0.01	Small

## Data Availability

De-identified data are available upon reasonable request from the corresponding author due to ethical and privacy restrictions. The dataset includes health-related information collected from human participants and cannot be shared publicly in compliance with GDPR and local data protection regulations. Summary materials, study instruments, and analytic code are openly available on the Open Science Framework (OSF) at DOI: 10.17605/OSF.IO/XRTSE.

## Supplementary Materials

The following supporting information can be downloaded at: https://www.mdpi.com/article/10.3390/healthcare13212706/s1, Table S1: Exact wording of items related to energy and fatigue dimensions; Table S2: Per-item missingness and paired N (N = 250).
