# Supplementary material for: Clinical and Symptom Profiles of Long-COVID Patients in Italy: A Cross-Sectional Analysis
Source: Healthcare (Basel). 2025 Oct 27;13(21):2706. doi: 10.3390/healthcare13212706 (PMC12610754; doi:10.3390/healthcare13212706)
Supplement: Supplementary file 1 [file healthcare-13-02706-s001.zip › healthcare-3895290-supplementary.pdf]

Supplementary Table S1. Exact wording of items related to energy and fatigue dimensions

Item label	Exact wording	Intended construct
**Muscle weakness**	“Have you experienced muscle weakness (loss of strength in the limbs or body)?”	Physical strength
**Fatigue/asthenia**	“Have you felt fatigue or asthenia (reduced energy or ability to sustain effort)?"	General energy level
**Excessive tiredness**	“Have you felt excessively tired or easily exhausted even after rest?”	Perceived exhaustion

Supplementary Table S2. Per-item missingness and paired N (N = 250)

Item /Variable	Missing n (%)	Paired N used
**Muscle/joint pain**	0 (0.0)	250
**Muscle weakness**	0 (0.0)	250
**Excessive tiredness**	0 (0.0)	250
**Fatigue/asthenia**	0 (0.0)	250
**Headache**	0 (0.0)	250
**Sociodemographic variables**	0-2 (0-0.8)	250
**Comorbidities and severity**	0 (0.0)	250

Note: No systematic missingness was detected; all core symptom comparisons were conducted on complete paired data (N = 250).
